# ICG-mediated fluorescence-assisted debridement to promote wound healing

**DOI:** 10.1371/journal.pone.0291508

**Published:** 2023-09-21

**Authors:** Gang Zhu, Benjian Gao, Jun Fan, Jianfei Chen, Song Su, Xiaoli Yang, Bo Li, Cheng Fang

**Affiliations:** 1 Department of General Surgery (Hepatobiliary Surgery), The Affiliated Hospital of Southwest Medical University, Luzhou, China; 2 Nuclear Medicine and Molecular Imaging Key Laboratory of Sichuan Province, Luzhou, China; 3 Department of Hepatobiliary Pancreatic and Splenic Surgery, Luzhou People’s Hospital, Luzhou, China; 4 Academician (Expert) Workstation of Sichuan Province, Luzhou, China; Brandeis University, UNITED STATES

## Abstract

The purpose of this study was to examine the efficacy of ICG-mediated fluorescence molecular imaging (FMI) in debridement of necrotic tissue. 96 wound-infected rats were randomly divided into control group, ICG group, excitation light (EL)group and FMI group for debridement of necrotic tissue (n = 24). (I) Control group: only debridement; (II) ICG group: ICG injection before debridement; (III) EL group: Debridement under EL; (IV) FMI group: Debridement guided by ICG-mediated FMI. On the 3rd, 6th, and 9th days, the wound tissues of the rats in each group were collected for histological examination, and the levels of serum interleukin-4 (IL-4) and interferon-γ (INF-γ) were analyzed. The wound healing rate, wound score and body weight of the rats in each group were followed up until the wound healed. The results showed that the infected wounds of the rats in the FMI group had significant fluorescence development. The level of serum IL-4 in the FMI group was higher than that in the other three groups on the 6th day (*p*<0.01), while the level of INF-γ was lower than that in the other three groups on the 6th and 9th day (*p*<0.05). The results of dynamic wound tissue H&E staining indicated that the wound healing in the FMI group was better than the other three groups. The in vivo follow-up results showed that the wound healing rate and wound score of the FMI group were better than the other three groups, and the growth of rats had no difference with the other groups. ICG-mediated FMI can achieve accurate imaging of necrotic tissue for debridement, and so can accelerate wound healing, which has good clinical application prospects.

## Introduction

Debridement is the removal of necrotic tissue and foreign body from the wound to expose the underlying viable tissue in an effort to promote and expedite wound healing. It forms an important component of the wound bed preparation in the management of chronic wounds [1,2]. Debridement rapidly transforms chronic wounds into easy-to-heal acute wounds that go through four overlapping stages: homeostasis, inflammation, proliferation, and remodeling. Studies have shown that during this process, several anti-inflammatory factors are produced either locally or systemically to participate in wound healing, such as typical interleukin (IL)-4 and interferon (IFN)-γ [3]. In addition, debridement can also remove biofilms produced by bacteria, which would enhance the resistance of bacteria to antibiotics and the body’s immune system, and finally prolong the wound healing time [4–7].

Surgical debridement is considered the gold standard for debridement. Even though being effective, it requires doctors to have superb surgical skills. However, it may not be enough because it has been reported that even an experienced surgeon could get more than 30% of normal tissue debrided, which is not beneficial for wound healing [8,9]. Therefore, precise debridement is essential for wound bed preparation. Current studies have shown that enzymatic debridement, maggot debridement and autolytic debridement have certain necrotic tissue selectivity, but they require a rigorous aseptic environment and a long-term treatment [10] and carry the risk of inducing allergic reactions [11,12]. Therefore, a more effective and selective method is necessary to be developed.

Indocyanine green (ICG) is a cyanine fluorescent dye approved by the U.S. Food and Drug Administration (FDA) with excitation and emission wavelengths of 778 and 830 nm in serum, respectively [13]. ICG is currently commonly used in the operating room in various settings including the detection of malignancies and metastatic lymph nodes, assessing hepatic anatomy and function, as well as assessment of vascular perfusion to minimize the risk of anastomotic leak [14–17]. Recently, our researchers discovered for the first time that ICG has the ability to specifically bind to necrotic cells and demarcate the boundaries of necrotic tissue by fluorescence molecular imaging (FMI) [18]. Therefore, FMI technology can become one of the important auxiliary means of debridement. In fact, optical molecular imaging is widely used in preclinical research and has made major breakthroughs [19,20].

In this study, based on the specific binding of ICG to necrotic cells, we successfully used Sprague-Dawley (SD) rats to establish a fluorescence development model of skin infection necrotic tissue. In addition, we evaluated the therapeutic effect of FMI technology-assisted debridement on a molecular level, pathological level, and skin wounds in vivo.

## Materials and methods

### Materials

ICG was purchased from China Dandong Pharmaceutical Co., Ltd., staphylococcus aureus was purchased from Shenzhen Yibaishun Technology Co., Ltd., rat IL-4 and INF-γ ELISA kits were purchased from Beijing Mindread Technology Co., Ltd., optical molecular imaging system Provided by Beijing Precision Digital Medical Technology Co., Ltd.

### Animals

96 female healthy SD rats were reared under suitable conditions for animal experiments. Animal experiments were performed according to the animal care guidelines and were approved by the Institutional Animal Care and Treatment Committee of Southwest Medical University (Luzhou, China) (Approval number: swmu20220002). All surgery was performed under sodium pentobarbital anesthesia, and all efforts were made to minimize suffering.

### Establishment of animal model of infection necrosis

#### Animal models and groups

After SD rats were anesthetized, a circular wound with a diameter of 2 cm was made on the back of each rat to expose the subcutaneous soft tissue. The activated Staphylococcus aureus suspension (0.5ml; 5*10^10^CFU/ml) was evenly injected into the exposed subcutaneous tissue of the rat. The 96 successfully modeled rats were randomly divided into 4 groups (n = 24) and all rats began debridement on the third day after infection. The Control group removed necrotic tissue empirically; In the ICG group, ICG (0.5 mg/kg) was injected through the tail vein before debridement, once every 4 hours, for a total of 3 times. The debridement method is the same as the Control group; The excitation light (EL) group was exposed to excitation light during debridement, and the debridement method was the same as that of the Control group; In the FMI group, ICG was injected into the tail vein, and the method was the same as that in the ICG group. 4 hours after the last injection of ICG, debridement was performed under the fluorescence navigation triggered by excitation light (The control group was used as a blank control, while the EL group and ICG group were used to exclude the influence of excitation light and ICG on wound healing, respectively). During experimental periods, rats were monitored 3 times per day for potential signs of suffering, mainly weight loss of more than 20% and significant changes in behavior, body posture or respiration. Rats with signs of pain or blood collection in the heart were euthanized by intraperitoneal injection of pentobarbital (120mg/kg) under deep anesthesia.

### Histopathological examination of rat wounds and detection of blood IL-4 and INF-γ inflammatory factors

6 rats in each group were randomly selected after debridement, and part of the wound tissue was collected on the 3rd, 6th, and 9th day, fixed with 10% formalin and stained with H&E. At the end of the wound tissue collection, rats were euthanized and 5 ml of cardiac blood was collected. Blood samples were centrifuged (10 min, 3500 r/pm) to separate the serum, which was stored at -80°C. The sample uses ELISA to detect the levels of the pro-inflammatory factor IL-4 and the anti-healing factor INF-γ.

### In vivo wound healing study

The changes of the wounds of the rats in each group were dynamically observed and the wound healing rate was calculated. Wound healing rate: (T-Tn) / T * 100%, T represents the initial wound area, namely T = 4π cm^2^, Tn represents the wound area in different observation periods, and when the wound healing rate reaches 95%, it is considered as Completely healed, and the body weight and wound scale of the rats in each group were measured at the same time (The scoring rules are shown in Table 1 [21]).

**Table 1:** Wound morphology scoring standard table.

wound condition	scores		
	0	1	2
Eschar formation	No	Little on edges	Thick
Odema/swelling	No swelling	Slight swelling	Heavy swelling
Fluid discharge	No discharge	Little discharge	Heavy discharge
Appearance of pus/slough	Nil	Slight presence	Heavy presence

### Statistical analysis

The experimental data were statistically analyzed by graphpad prism 6.0 software, and the data were expressed by mean ± standard deviation. One-way ANOVA was used for comparison between multiple groups, and LSD-t test or Mann-Whitney U rank-sum test was used for comparison between groups. *p* < 0.05 was considered to be statistically significant.

## Results

### Application of fluorescence navigation in rat necrosis model

#### Fluorescence imaging of infected wounds in rats

**Fig 1:**
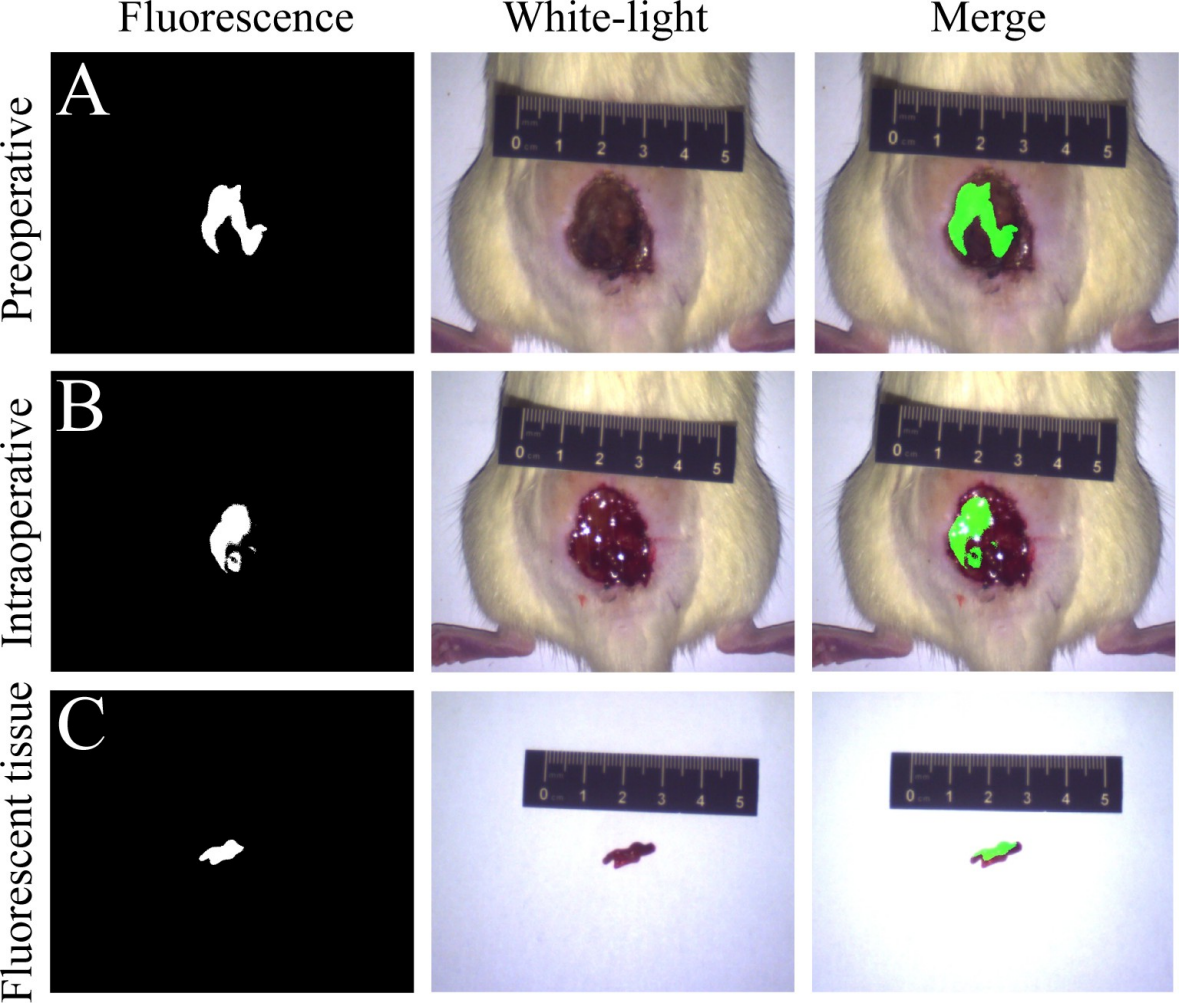
Resection of infected necrotic crust and necrotic tissue in rats under fluorescent navigation. (A) Fluorescent image of crusts on infected wounds; (B) Fluorescent tissue under the necrotic crust; (C) The fluoroscopic tissue was removed as completely as possible under the intraoperative fluoroscopic navigation.

Fig 1 shows the fluorescence images of the wounds of the rats in the FMI group. Using the fluorescence imaging device, significant fluorescence expression can appear in the infected wound of rats in FMI group (Fig 1A). After the necrotic scab on the infected wound surface of the rats in the FMI group was removed, the underlying tissue was still fluorescently visualized, and this part of the tissue could not be identified by the naked eye whether it was necrotic (Fig 1B). Finally, all fluorescently visualized tissues were cleared under the fluorescence image navigation (Fig 1C).

#### Histological examination of rat fluorescence imaging tissue

**Fig 2:**
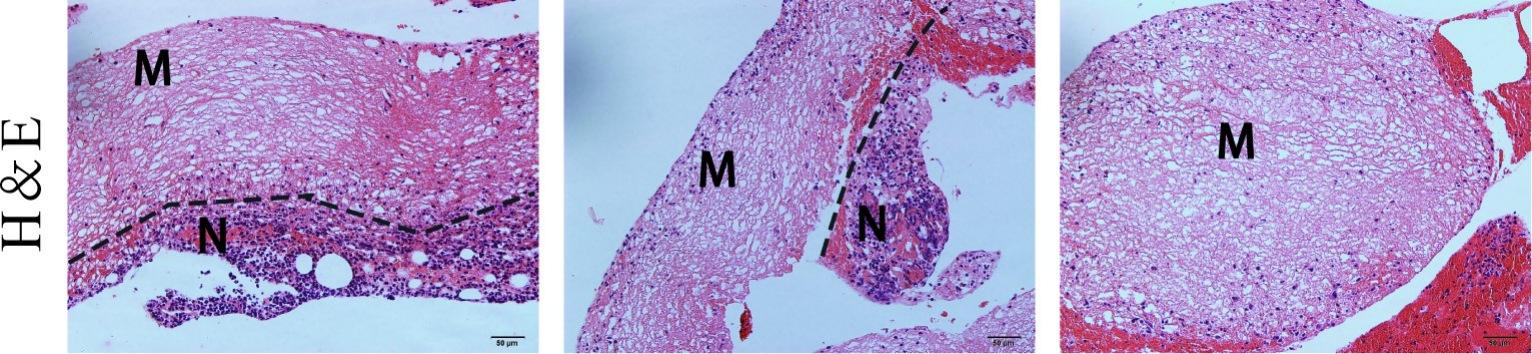
H&E staining pictures of the rat fluorescent tissue, the dotted line represents the boundary between necrotic tissue and inflammatory cell infiltration tissue, M represents the necrotic area, and N represents the non-necrotic area. (scale bar, 50 μm).

As shown in Fig 2, a large area of tissue structure was disorganized, no normal cell structure, and a large number of cell nuclei disappeared under the microscope, showing a homogeneous red staining phenomenon, which formed a clear boundary with the tissue infiltrated by inflammatory cells, which is consistent with the fluorescence imaging range shown in Fig 1C.

#### Histological examination at different time periods

**Fig 3:**
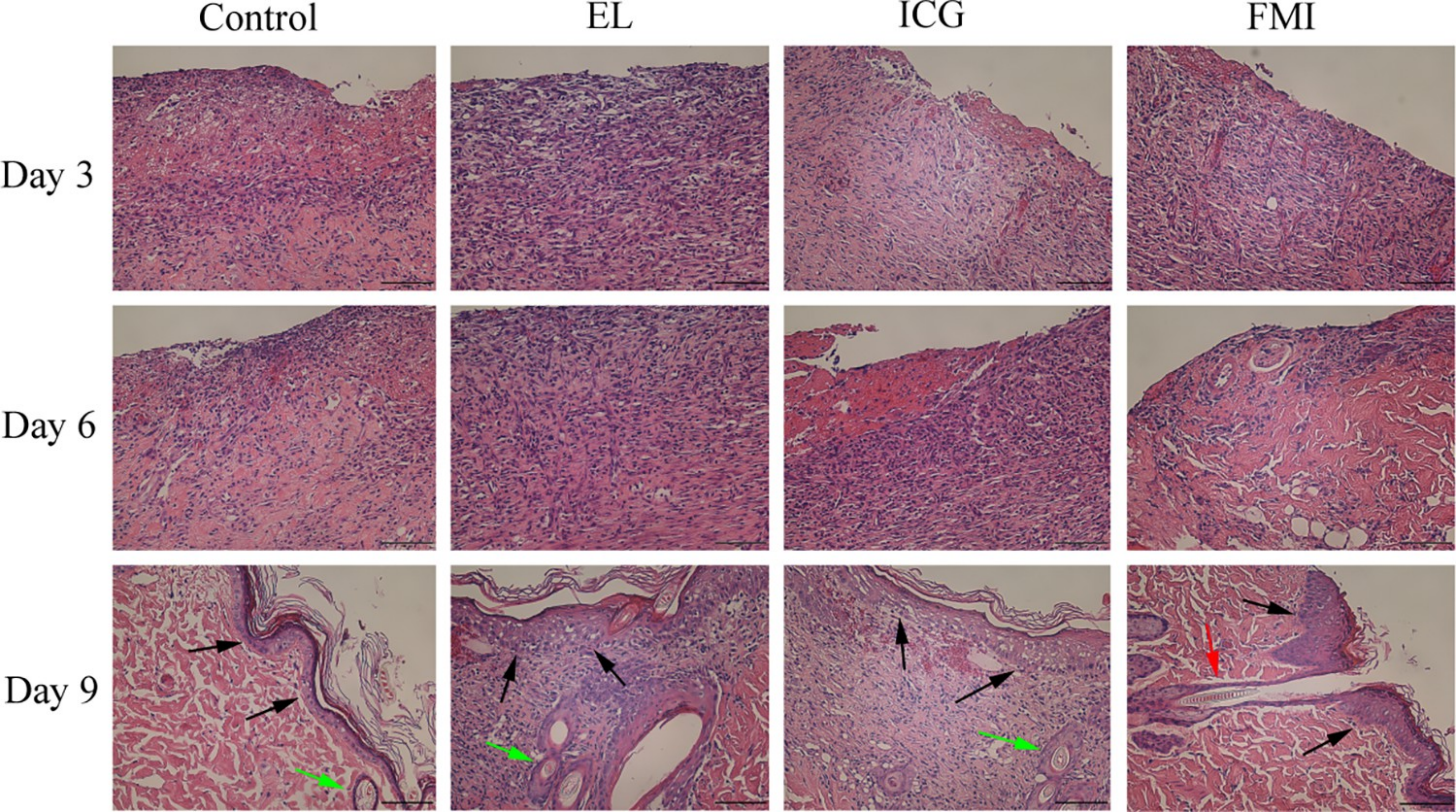
H&E staining results of wound tissue in the Control group, EL group, ICG group and FMI group on the 3rd, 6th, and 9th days after the operation. The black arrows point to the epidermoid tissue, The green arrows point to the immature hair follicles, The red arrow points to mature hair follicles (scale bar 100 μm).

Fig 3 shows H&E staining images of wound tissue in four groups of rats on days 3, 6, and 9. On the 3rd day, the wounds of the four groups of rats lacked epidermoid tissue coverage and were infiltrated by a large number of inflammatory cells. On day 6, a small amount of epidermoid tissue and new blood vessels appeared in the FMI group, and the number of inflammatory cell infiltration decreased. The wounds of the other three groups of rats showed no significant change compared with the 3rd day, and the wounds were still infiltrated by a large number of inflammatory cells and lacked epidermoid tissue coverage. The H&E results of the wound tissue on the 9th day showed that the wounds of the Control group, the EL group and the ICG group were covered with epidermal tissue structures, and a small number of primary hair follicles were seen, but the new epidermal structure was disordered and there were still scattered inflammatory cell infiltration underneath. The coverage of epidermoid tissue in the FMI group was better than that in the other three groups, the infiltration of inflammatory cells almost disappeared, and mature hair follicles and subcutaneous glands could be seen in the deep dermis.

#### IL-4 and INF-γ levels

**Fig 4:**
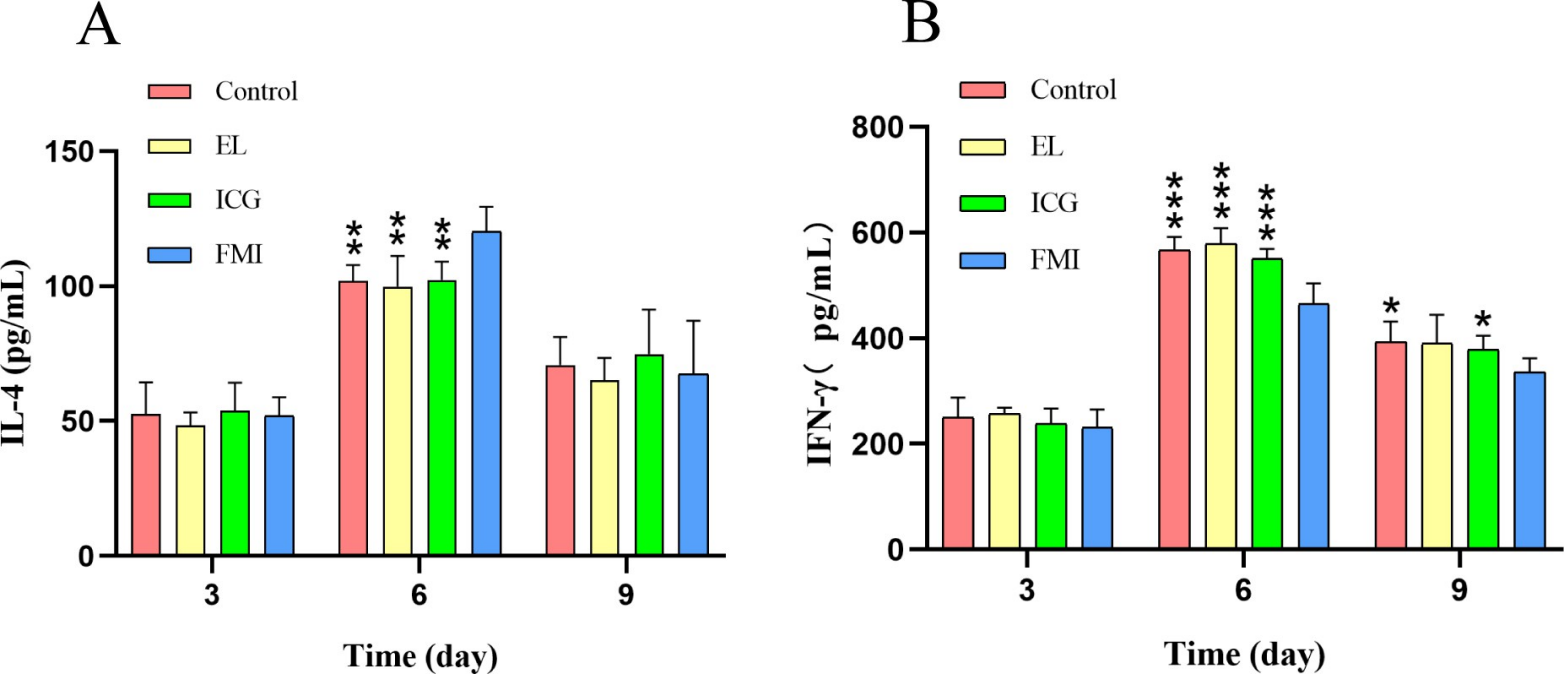
ELISA results for IL-4 and INF-γ, *p < 0.05, **p < 0.01, *** *p* < 0.001 (LSD t-test).

Fig 4A shows the changes in serum IL-4 levels of four groups of rats on days 3, 6, and 9. The results showed that on the 6th day, the serum IL-4 levels of the rats in the Control group, EL group, ICG group and FMI group were 101.93±5.94 (pg/ml), 99.76±11.56 (pg/ml), 102.32± 6.75 (pg/ml) and 120.24±9.17 (pg/ml), among which the serum IL-4 levels of the FMI group was higher than that of the other three groups, and the difference was statistically significant (*p*<0.01). However, on days 3 and 9, there was no statistical difference between the four groups.

Fig 4B shows the changes of INF-γ levels in the serum of four groups of rats on days 3, 6, and 9. The results of ELISA analysis showed that the serum INF-γ levels of the rats in the Control group, EL group, ICG group and FMI group were 566.24±25.75 (pg/ml), 579.28±29.10 (pg/ml), 551.33±17.67 (pg/ml), and 464.77±38.92 (pg/ml), respectively. The FMI group was less than the other three groups, and the difference was statistically significant (*p*<0.001). On the 9th day, the levels of INF-γ in the four groups were 393.77±38.06 (pg/ml) in the Control group, 390.52±53.60 (pg/ml) in the EL group, 378.24±27.02 (pg/ml) in the ICG group and 336.21±26.30 (pg/ml) in the FMI group. the INF-γ in the FMI group was lower than Control group and ICG group. (*p*<0.05).

#### In vivo wound healing

**Fig 5:**
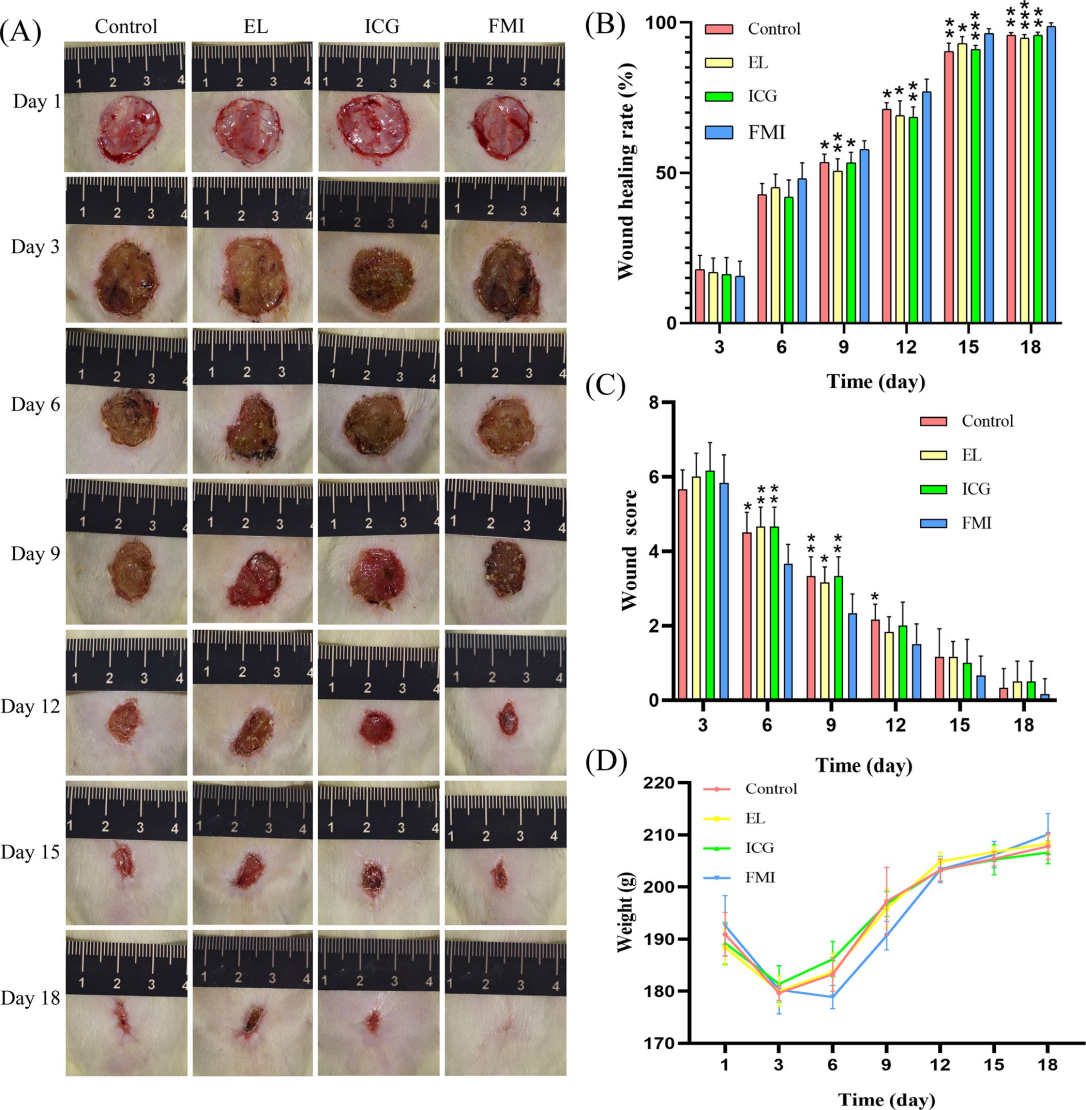
(A) Pictures of wound healing of four groups of rats over time; (B) The percentage of wound closure rate and healing time in the four groups of rats. (C) Changes of wound scores in four groups over time. (D) Changes of body weight of four groups of rats with time, *p < 0.05, **p < 0.01, *** *p* < 0.001 (LSD t-test).

Fig 5A shows the dynamic change images of wound healing in the four groups of rats. On the 3rd day, the wounds of the rats in each group had a similar degree of infection, and at this moment, the necrotic tissue of the wounds of the rats in each group was removed according to the experimental design. On the 6th day, the wounds of the rats in the four groups reappeared with scabs. The wounds of the FMI group were relatively dry, while the wounds of the other three groups still had purulent secretions. On the 9th, 12th, 15th, and 18th days, the wounds of the four groups of rats continued to shrink, and the wounds of the rats in each group were not infected. In general, the healing of the FMI group was better than that of the other three groups.

Fig 5B shows the wound healing rate of four groups of rats in different observation periods. From the 9th day, the wound healing rate of the FMI group was higher than that of the other three groups. Meanwhile, on the 15th day, the FMI group (96.25%±1.72%) reached the standard of complete healing, while that of the control group (90.33% ± 2.84%), ICG group (91.04% ± 1.35%) and EL group (92.92% ± 2.40%) did not achieve.

Fig 5C shows the changes in the wound scores of the four groups of rats. The wound scores of the rats in the FMI group (3.67±0.52) were lower than those of the other three groups (Control group 4.5±0.55, EL group 4.67±0.52, ICG group 4.67±0.52) on the 6th day. On the 9th day, the wound scores of the rats in the FMI group (2.33±0.52) were also lower than those of the other three groups.

Fig 5D shows the body weight changes of the four groups of rats in 18 days, except on the 6th and 9th days (*p*<0.05), the FMI group was smaller than the other three groups, and there was no difference between the four groups at other time points. No rats died during the observational study.

## Discussion

Wound healing proceeds in three sequential and overlapping phases beginning with inflammation, proceeding to granulation tissue formation, and finally matrix formation and remodeling [22]. This process involves highly coordinated interactions between dermal and epidermal cells and is regulated by multiple chemical mediators. It is well known that the healing process of wounds is affected by various factors both systemically and locally, especially infection of wounds leads to delayed healing and worse overall outcomes such as excessive scarring [23,24]. Debridement, as an important way to control infection, plays an important role in promoting wound healing. Various debridement methods, including surgical, autolytic, osmotic, enzymatic, biological and mechanical, have been widely used in clinical practice. Here, for the first time, we used ICG-mediated FMI-assisted surgical debridement of necrotic tissue and explored its effectiveness in a rat model of infected necrosis.

ICG enables a visual assessment of perfusion owing to its binding to plasma lipoprotein (LP) and ability to emit fluorescence when treated with a certain wavelength laser. It has found multiple applications in various general surgical procedures to assess perfusion of anastomoses, identification of critical structures and guide resection of hepatic carcinoma [25–27]. However, unlike in liver cancer tissues, the necrotic affinity mechanism of ICG stems from its special molecular structure. ICG consists of two polycyclic moieties (benzindoletricarbocyanins) that are very lipophilic and linked by a carbon chain. The combination of sulfate groups makes ICG molecules have a certain water solubility. This complex molecular structure leads to amphiphilic properties [28]. The ICG-LP complex is too large to penetrate normal blood vessel walls, unless vascular permeability increases due to certain disorders such as an inflammation or trauma [28,29]. Our researchers speculate that when the ICG-LP complex is freed from the blood vessel into the necrotic tissue space, the LP shields the hydrophilic end of ICG, ICG-LP shows enhanced affinity for hydrophobic groups. Moreover, the ruptured phospholipid bilayer exposes the hydrophobic tail of phospholipids, so ICG-LP may exhibit affinity for the phospholipids in necrotic cells [18]. Based on previous studies, we further investigated the efficacy of ICG FMI-assisted debridement in the treatment of necrosis.

In this study, due to the rapid metabolism of ICG in vivo, we chose to use intermittent injection to improve the probability of ICG entering the necrotic tissue area (One-time slow pumping of ICG can be used in clinical practice). On the wounds of rats injected with ICG, we obtained significant fluorescence images, including necrotic crusts on the surface and some necrotic soft tissue under the crusts (Fig 1), and the microscopic level also delineated the boundaries of necrosis (Fig 2), which is consistent with our previous studies [18]. Incomplete phospholipid bilayers of necrotic cells as targets bind to ICG-LP to realize FMI of necrosis.

The generation of cells in skin tissue, changes from cell structure and changes from tissue structure can intuitively reflect the degree of skin healing. As can be seen from Fig 3, the wound tissue of the FMI group healed faster than the other three groups, and the appearance of epidermoid tissue and mature hair follicles was faster than the other three groups. Reepithelization is critical for optimal wound healing not only because it re-forms the cutaneous barrier but also because of its role in wound contraction [30]. In vivo wound-healing studies also showed that the wounds of rats debridement assisted with fluorescence were better than those without fluorescence assistance (Fig 5).

The inflammatory stage of wound healing commonly takes place from the first minutes to several days [31]. Our histological results showed that at day 6 and day 9, the number of inflammatory cells in the FMI group was significantly lower than the other three groups (Fig 3). This result indicates that fluorescence-assisted debridement improves the removal rate of wound necrotic tissue, which can accelerate and shorten the duration of the inflammatory phase. Acceleration of the inflammatory stage could partly explain the significant difference in wound area after day 9 (Fig 5A and 5B).

As previously stated, the wound healing response is regulated by a large quantity of cytokines secreted by the associated cells [32]. Two important cytokines, IL-4 and IFN-γ, were analyzed in our study, and our results showed that serum IL-4 levels measured on day 6 after fluorescence-assisted debridement in infected rats were significantly higher than those of the other three groups, while IFN-γ levels were lower on days 6 and 9 than those of the other three groups. The reasons for this result are related to the control of wound infection and the degree of wound healing. IL-4 is an important cytokine involved in anti-inflammatory processes, cell growth, immune regulation [33]. Evidence for the ability of IL-4 to induce the polarization of macrophages to M2 with anti-inflammatory capabilities is increasing [34,35]. In addition, IL-4 accelerates the synthesis of proteins important to the healing process, such as fibronectin, collagen I and II [36]. IFN-γ is mainly produced by NK cells and CD4^+^ Th1 cells and has multiple effects on macrophages, NK cells, and T lymphocytes [37]. In contrast, In vitro studies have shown that INF-γ has the effect of inhibiting the synthesis of collagen fibers by fibroblasts [38,39]. The changes of the two inflammatory factors jointly indicated that the wound healing speed of the rats in the FMI group was faster than that in the other experimental groups.

In summary, our results show that ICG can accurately identify necrotic tissue, and debridement assisted by fluorescence can promote the healing of infected wounds in rats. In view of the biological safety of ICG, debridement assisted by fluorescence is expected to be widely used in clinical practice.

## Supporting information

S1 Table IF-4. (XLSX)

S2 Table IFN-γ. (XLSX)

S3 Table Wound healing rate. (XLSX)

S4 Table Changes of wound scores in four groups. (XLSX)

S5 Table Changes of body weight of four groups. (XLSX)
